# Supplementary material for: Deep learning-based real-time seizure detection and multi-seizure classification on pediatric EEG
Source: Front Neurol. 2026 Feb 23;17:1726258. doi: 10.3389/fneur.2026.1726258 (PMC12968684; doi:10.3389/fneur.2026.1726258)

**Supplementary Figure 1.** Area Under the Curve (AUROC) plots for binary seizure detection by different models and sliding window sizes (4 s figures (a)-(d), 12 s (e)-(h)): (a), (e) CNN2D+LSTM, (b), (f) ResNetshort+LSTM, (c), (g) ResNetshort+Dilation+LSTM, (d), (h) MobileNetV3short+LSTM.


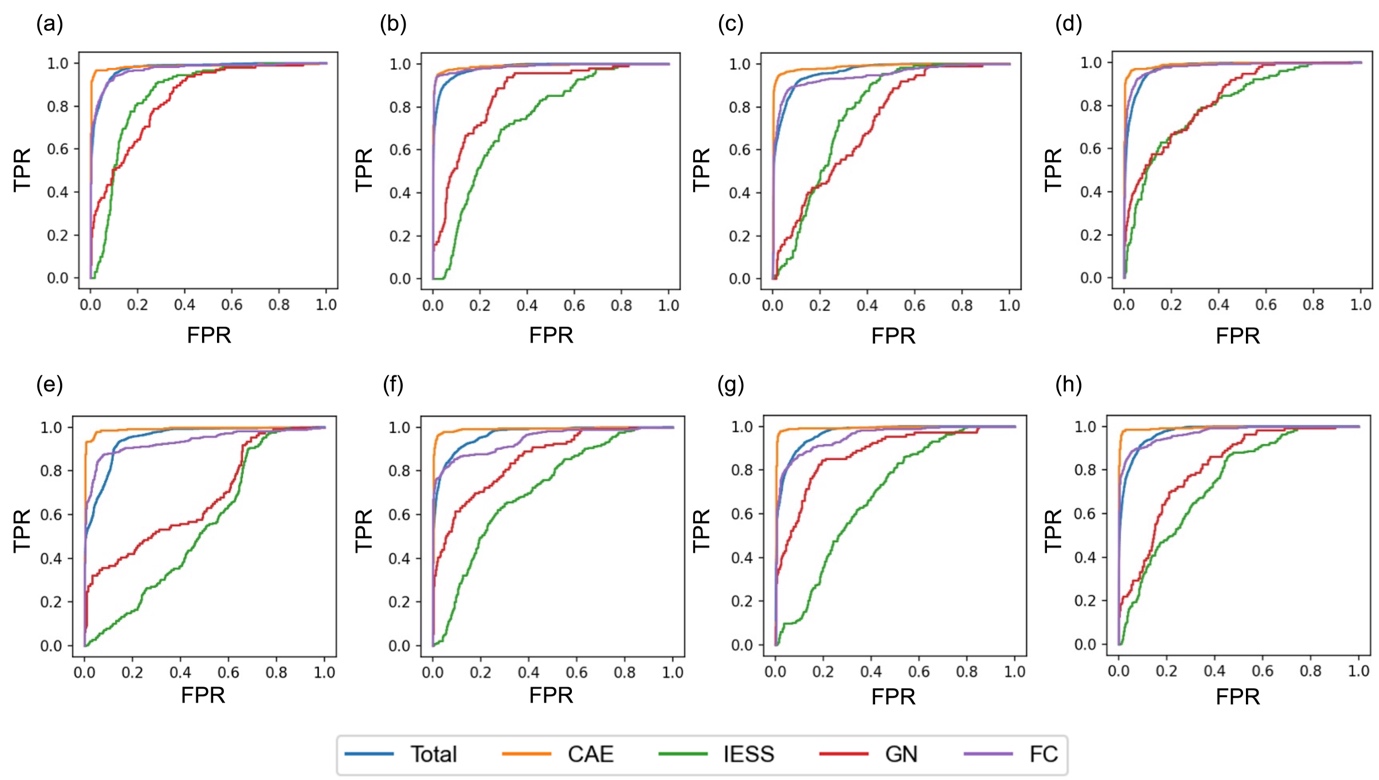


**Supplementary Figure 2.** Area Under the Curve (AUROC) plots for multi-class seizure classification by different models and by feature extractor (raw signal (a)-(d), power spectral density (frequency bands) (e)-(h)): (a), (e) CNN2D+LSTM, (b), (f) ResNet50, (c), (g) MobileNetV3, (d), (h) DenseNet.


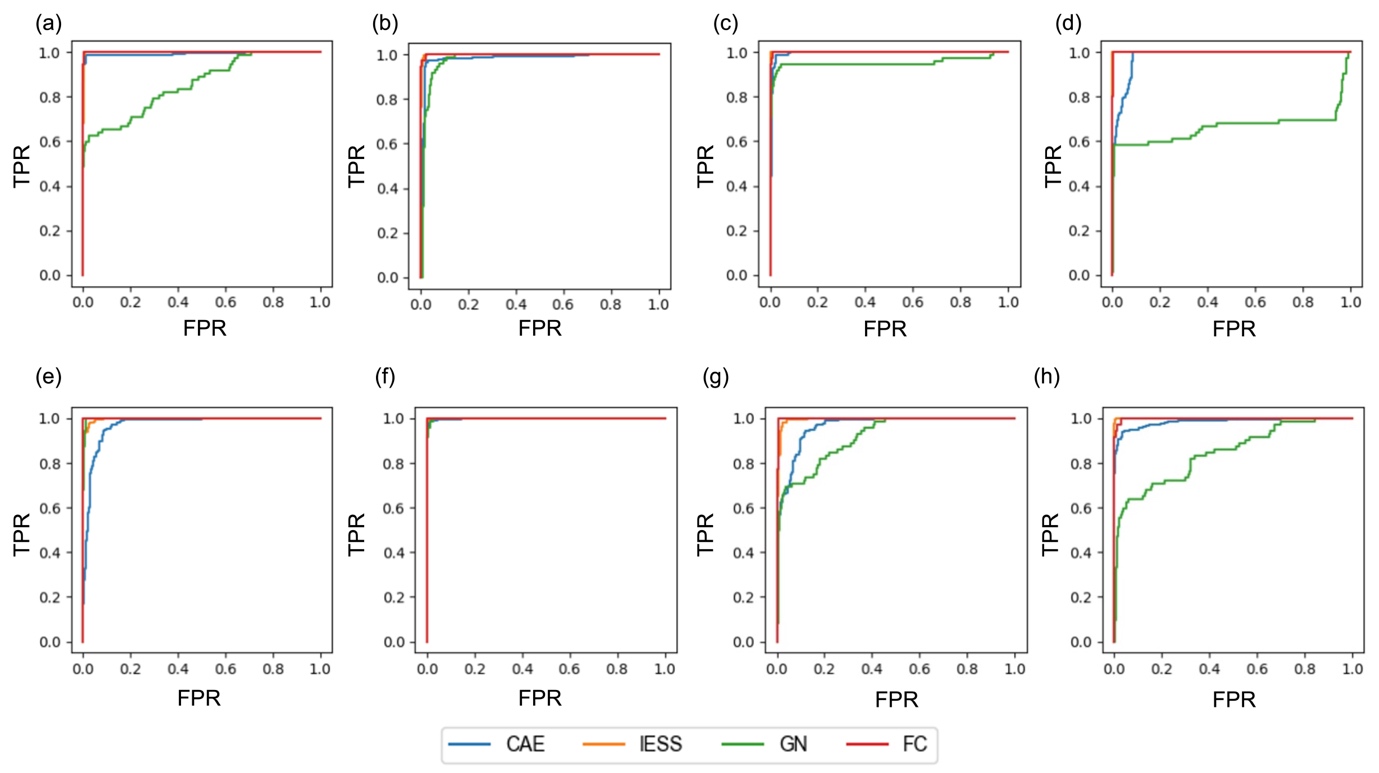

Supplement: Supplementary file 1 [file Table_1.docx]
