# Supplementary material for: Deep learning-based real-time seizure detection and multi-seizure classification on pediatric EEG
Source: Front Neurol. 2026 Feb 23;17:1726258. doi: 10.3389/fneur.2026.1726258 (PMC12968684; doi:10.3389/fneur.2026.1726258)
Supplement: Supplementary file 2 [file Table_2.docx]

**Supplementary Table 1.** Model parameter size for detection and classification modules.

| Module type | Model architecture | Parameters (M) |
| --- | --- | --- |
| Detection | CNN2D + LSTM | 1.54 |
|  | ResNet-short + LSTM | 3.94 |
|  | ResNet-short + Dilated + LSTM | 3.94 |
|  | MobileNetV3-short + LSTM | 1.36 |
| Classification | CNN2D + LSTM | 12.90 |
|  | ResNet50 | 16.73 |
|  | MobileNetV3 | 4.20 |
|  | DenseNet | 0.37 |

Detection module parameter counts were estimated from previously reported model sizes and expressed in million parameters (M).
